# The importance of integrated care in the management of dysphagia among homebound older patients with heart failure: a narrative review

**DOI:** 10.3389/froh.2025.1738507

**Published:** 2026-01-12

**Authors:** Masahiko Okubo, Tomohisa Ohno, Hideo Sakaguchi, Takeshi Kikutani, Shoichiro Kokabu

**Affiliations:** 1 Department of Dentistry and Oral Surgery, Ongata Hospital, Hachioji, Japan; 2 Tama Oral Rehabilitation Clinic, The Nippon Dental University, Iidabashi, Japan; 3 Department of Dentistry, Ryohoku Hospital, Hachioji, Japan; 4 Division of Biochemistry, Kyushu Dental University, Kitakyushu, Japan; 5 Oral Medicine Innovation Center, Kyushu Dental University, Kitakyushu, Japan

**Keywords:** end-of-life care, home care, nutritional management, older adults, quality of life, swallowing rehabilitation

## Abstract

**Introduction:**

Older homebound patients frequently present with dysphagia and heart failure, two interrelated geriatric syndromes that create a negative cycle resulting in aspiration pneumonia, malnutrition, hospital readmissions, poor prognosis, and diminished quality of life (QOL). In this review, we highlight the importance of integrated care and propose practical approaches to managing these conditions in home care settings.

**Methods:**

We conducted a narrative review of the literature addressing the association between dysphagia and heart failure in older adults, with a focus on studies and guidelines relevant to home care. Key themes regarding pathophysiology, clinical challenges, and multidisciplinary strategies for comprehensive management were identified.

**Results:**

Heart failure can impair swallowing function through circulatory insufficiency, sarcopenia, and overall frailty. Conversely, dysphagia can worsen heart failure through repeated aspiration events and poor nutritional intake. Compared with hospital environments, home care is resource-limited, leading to delays in assessment and intervention. Evidence suggests that integrating dysphagia management into heart failure care, even in such settings, may help mitigate the negative cycle. Involving multidisciplinary teams—including physicians, nurses, dentists, dietitians, and speech-language-hearing therapists—can optimize circulatory dynamics, nutritional status, oral function, and safe oral intake based on individual risk assessment.

**Discussion:**

Integrated and multidisciplinary care is essential for managing older homebound patients with comorbid heart failure and dysphagia. Tailored approaches that consider disease stage and patient and caregiver preferences can enhance safety, reduce readmissions, and maintain dignity and QOL. This review underscores the urgent need for comprehensive care models addressing both conditions simultaneously in resource-limited home care environments.

## 1 Introduction

In a super-aged society, dysphagia is recognized as a critical issue affecting the QOL and prognosis of homebound patients (1–3). It is one of the geriatric syndromes, which develops and progresses owing to a combination of factors, including aging, multimorbidity, and physical decline. In hospital-based cohorts of patients with heart failure (HF) aged 60 and above, the prevalence of dysphagia at admission was 23.6% (range, 14.1%–32.9%). Moreover, in these hospitalized cohorts, the condition persisted until discharge in 9.4% of patients (4). Additionally, in older patients with HF, various factors such as circulatory insufficiency, congestion, systemic sarcopenia, and dyspnea contribute to the deterioration of swallowing function, which in turn worsens prognosis through aspiration pneumonia and malnutrition (5, 6). HF itself is also closely linked to geriatric syndromes, necessitating a multifaceted approach to its management in older adults.

In hospital-based studies of acute HF admissions, approximately 23% of patients have been reported to have coexisting dysphagia (7). In hospitalized populations, dysphagia is associated with prolonged hospitalization, limited discharge options, and an increased in-hospital mortality rate (8, 9).

Recently, home care for older patients with HF has been increasingly promoted, leading to a growing number of cases transitioning from acute care in hospitals to comprehensive community-based care (10). However, compared with hospital settings, home care is limited in both medical and human resources, which can delay the assessment and intervention for conditions such as dysphagia. This delay increases the risk of aspiration pneumonia and nutritional deterioration, potentially resulting in hospital readmission or the inability to continue home care (11, 12). Therefore, an approach in which the presence of dysphagia is considered is essential in the home management of HF. Without adequate attention to swallowing function, the effectiveness of HF treatment itself may be compromised.

In this review, an overview of the pathophysiological relationship between dysphagia and HF was provided, along with strategies for nutritional management and QOL improvement for patients, practical approaches to assessment and intervention in home care, and the continuum of care up to end-of-life management. The significance of an integrated approach was highlighted and future challenges were discussed. Additionally, the practical value of meal rounds and simple screening tests as methods for observing eating behavior were explored, offering actionable insights for the care of older patients who are homebound.

## 2 Methods

To conduct a comprehensive review, we searched PubMed as the primary database. We prioritized applicability to home care and explicitly distinguished evidence derived from hospital-based studies vs. home care/community settings when summarizing findings. We used systematic Boolean combinations (AND/OR), with database-specific syntax adjusted as needed. Representative search strings included the following combinations: (1) (dysphagia OR swallowing disorders) AND (heart failure OR HF OR cardiac failure); and (2) (dysphagia OR swallowing) AND (elderly OR older adults). Additional combinations were used to capture home-care and nutrition-related themes: (3) (home care OR home-based care OR homebound) AND dysphagia; (4) (oral intake OR oral feeding) AND quality of life; and (5) (malnutrition OR undernutrition) AND heart failure. When appropriate, relevant articles were additionally checked in Scopus and Google Scholar using analogous combinations. The initial search identified 587 records (PubMed, *n* = 342; Scopus, *n* = 156; Google Scholar, *n* = 89). After removing duplicates, 421 records remained. Title/abstract screening yielded 82 articles for full-text assessment (excluding pediatric populations, non-cardiogenic dysphagia, editorials/letters, articles not relevant to home care, and out-of-scope languages). All 82 full texts were evaluated for applicability to home/community care, relevance to HF–dysphagia interactions, and clinical/theoretical importance, resulting in 71 included references in the final narrative synthesis. Inclusion criteria were as follows: studies involving older adults (typically aged 65 and above); studies addressing dysphagia, heart failure, and their interaction; articles discussing home care, nutrition, sarcopenia, and oral intake in the context of dysphagia; and peer-reviewed clinical studies and review articles written in English or Japanese. Exclusion criteria were as follows: studies not involving elderly or home care populations; articles focusing solely on pediatric or neurological dysphagia without cardiac comorbidity; and non-peer-reviewed articles, editorials, and letters to the editor. We focused on articles published in the past 10 years (2014–2024). However, we also included selected pre-2014 seminal works when they established foundational concepts or standard clinical frameworks that remain widely accepted and frequently cited, or when newer evidence for core concepts was limited.

## 3 The interaction between dysphagia and heart failure

Dysphagia and HF have a complex, bidirectional relationship, and a detailed understanding of their pathophysiology is crucial for formulating appropriate treatment strategies. One factor contributing to the worsening of dysphagia in HF is systemic edema caused by circulatory insufficiency, congestion, and pulmonary edema, which may affect the pharyngeal mucosa and potentially lead to incomplete closure of the epiglottis and vocal cords (13). Additionally, the mechanism by which severe enlargement of the left atrium compresses the esophagus, resulting in what is known as cardiogenic dysphagia, has been reported for some time. In cases of significant left atrial enlargement, such as in mitral valve disease or dilated cardiomyopathy, the esophageal diameter is physically narrowed, making it difficult for bolus passage during swallowing (14–16).

Chronic HF promotes sarcopenia and cachexia and accelerates the decline in activities of daily living (ADL) and motor function due to malnutrition and fatigue (17, 18). The oral muscles involved in chewing and swallowing, including the perioral muscles, tongue, and pharyngeal muscles, are no exception, and muscle weakness may cause insufficient bolus formation and pharyngeal contraction (19). Furthermore, dyspnea and orthopnea, commonly observed in patients with HF, can disrupt the coordinated movements of breathing and swallowing. Swallowing temporarily suppresses breathing; however, it is believed that, in the presence of respiratory distress, the ability to adequately cease breathing is impaired, increasing the risk of aspiration (8, 20, 21).

**Figure 1:**
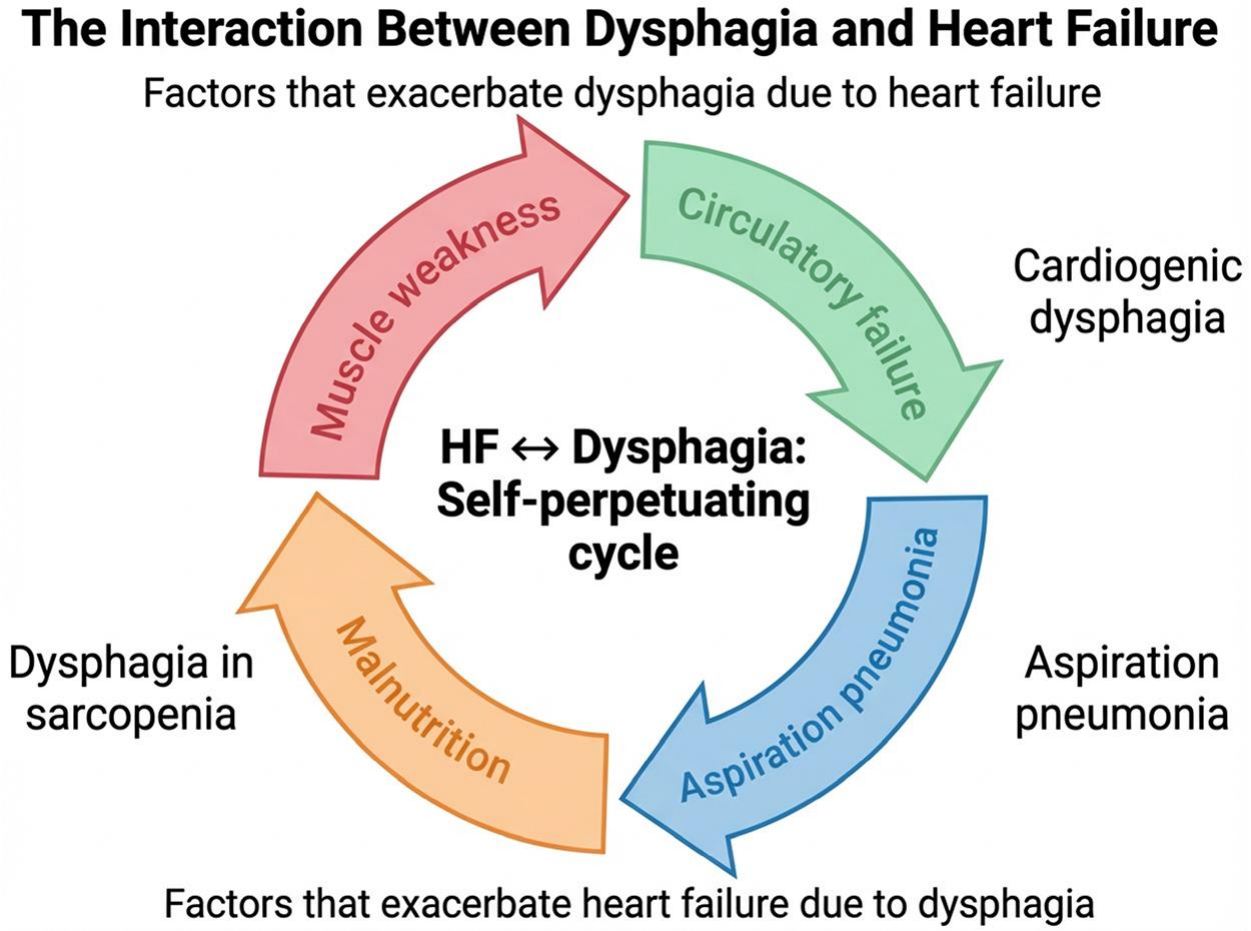
The interaction nnbetween dysphagia and heart failure. This schematic illustrates a bidirectional relationship between heart failure (HF) and dysphagia. HF may contribute to dysphagia via circulatory failure and muscle weakness, whereas dysphagia may exacerbate HF through aspiration pneumonia and malnutrition, forming a self-perpetuating cycle.

Conversely, dysphagia can worsen HF through several mechanisms (Figure 1). First, the worsening of malnutrition is a major contributing factor. Oral intake reduction and excessive dietary restrictions to prevent aspiration increases the risk of weight loss and decline in various nutritional markers, which in turn aggravates the cachexia associated with HF (22, 23). Second, aspiration pneumonia resulting from dysphagia raises cardiac load through infection-related stress and leads to hypoxemia from respiratory failure and an increase in inflammatory mediators, further exacerbating HF (24). Furthermore, swallowing-modified diets aimed at reducing aspiration risk may potentially worsen HF because they have high fluid content. This negative cycle suggests that the traditional approach of treating dysphagia and HF separately and independently may not sufficiently address the problem. Given the interdependent nature of the pathophysiology of both conditions, it is crucial to provide integrated care that combines home care, rehabilitation, and pharmacological treatment. In ICU and other acute hospital settings, conventional strategies to manage dysphagia include behavioral swallowing treatment such as modifying the texture of diet/fluids. In addition, compensatory techniques, including elevating the head while lying on the bed, and dental brushing with antiseptic rinse and suctioning, are implemented to minimize risk and further complications. Postural and positioning changes may provide some therapeutic benefits as well. However, these approaches may not address the underlying problems with swallowing function (25). In contrast to ailments of the central nervous system, HF does not directly affect the somatosensory system or swallowing center. Nonetheless, improving functional oral intake during acute care for patients with HF is independently associated with physical function, nutritional status, and maximum tongue pressure (26). Therefore, interventions aimed at strengthening swallowing-related muscle groups and improving overall physical function (including frailty) may help mitigate dysphagia associated with heart failure.

## 4 Evaluation of swallowing function, practical meal observations, and dysphagia management for improved QOL

### 4.1 Swallowing screening and evaluation in home care

Professional evaluations, such as fiberoptic endoscopic evaluation of swallowing and videofluoroscopic swallowing study (VFSS) are commonly used to assess swallowing function. However, these procedures cannot be performed in home care settings. In home care settings, there are often limitations in evaluating swallowing function caused by the lack of advanced equipment available in hospital facilities. Therefore, simple and practical screening tests and meal observations become essential. For example, the Repetitive Saliva Swallowing Test (RSST), the modified water swallow test, the Eating Assessment Tool-10 (EAT-10), and the Saku-Saku Test (which evaluates jaw mobility and chewing feasibility by observing up–down and circular jaw movements with the mouth lightly closed) can be implemented during home visits, helping to identify aspiration risk early (27–29). These simple screening tools can be administered during home visits by trained professionals (e.g., nurses, speech-language pathologists, or dentists/dental hygienists), and may also be performed by caregivers/care staff after appropriate instruction. Screening is recommended at enrollment/initial assessment (especially after discharge for HF exacerbation), repeated when clinical changes occur, and otherwise monitored periodically (e.g., every 1–3 months depending on risk and goals of care). A positive screen should prompt clinical evaluation and referral to swallowing specialists, with instrumental assessment (e.g., portable endoscopic evaluation) when indicated, followed by individualized management aligned with patient preferences. In Japan, portable endoscopic devices, particularly for Fiberoptic Endoscopic Evaluation of Swallowing (FEES), are increasingly being adopted for swallowing assessments at the patient's bedside or during home visits (30).

### 4.2 Practical meal observations (meal rounds)

Recently, increasing attention has been on a method called meal rounds, which involves multidisciplinary observation of actual mealtime situations (31). By comprehensively observing aspects such as the procedure and posture during meal assistance, oral hygiene, and control of food intake, it is possible to identify individual swallowing patterns and issues that cannot be fully understood through mere numerical assessments.

The impact of dysphagia on the QOL of patients and caregivers should not be overlooked. The loss of joy in eating and limitations on social participation are factors that reduce a patient's motivation, as well as physical and mental function. In fact, the Swallowing QOL questionnaire is disease-specific and is used to assess the subjective QOL assessment of individuals with dysphagia. It has been shown that dysphagia causes multidimensional declines in QOL, including physical, psychological, and social aspects, with the Japanese version showing high reliability (32–35).

### 4.3 Quality of life (QOL) and caregiver burden

Meal assistance can be highly stressful for family caregivers in home care. Anxiety about aspiration or choking, together with restrictions on social activities, may increase caregiver burden, reduce QOL, and contribute to fatigue. This burden can make continuing home care challenging (27). Therefore, coordinated HF–dysphagia management, together with multidisciplinary support for families, is essential to improve QOL for patients and caregivers.

In home meal guidance and nutritional counseling, initially setting the appropriate energy and protein intake based on the patient's oral intake capacity should be considered (36, 37). Following this, meal texture (such as soft food, pureed food, or thickened liquids) should be finely adjusted to reduce aspiration risk, stabilize food intake, and prevent malnutrition (38, 39). If adjusting the meal texture results in a decrease in effective nutritional intake, methods to increase nutritional content, such as using gelling agents and avoiding excessive dilution, should be explored. In some cases, short-term or supplementary enteral or peripheral parenteral nutrition may be used, but maintaining oral intake as much as possible is believed to improve QOL. Particularly, the loss of the joy of eating can lead to depressive symptoms, decreased motivation in older patients, and, ultimately, reduced adherence to HF treatment. Therefore, considering personal preferences, seasoning, and other psychosocial factors is recommended when supporting these patients (40–42). For patients with HF who have dysphagia, the challenge lies in balancing oral intake, adjusting the appropriate fluid amount, and reducing the risk of aspiration. Regularly evaluating circulatory and respiratory conditions, adjusting meal textures, and incorporating oral nutritional supplements (ONS) as needed are essential for flexible management.

## 5 Management and intervention strategies for dysphagia associated with heart failure

Management and intervention strategies for dysphagia are tailored as per the patients' clinical characteristic and history. Patients with pre-existing dysphagia at the time of discharge are typically identified through in-hospital assessments, such as VFSS or FEES, prior to transition to home care. For such patients, it is essential that structured dysphagia management begins immediately after discharge. Home care teams should implement individualized care plans that include texture-modified diets, oral hygiene protocols, postural modifications, and nutritional interventions. Tools such as RSST, modified water swallow tests, and meal rounds are used during early home visits to reassess the patient's swallowing ability and confirm the safety of oral intake in the home context. Follow-up should be conducted regularly, especially during the initial 2–4 weeks, to adapt the care plan according to physical and functional changes.

For patients who develop dysphagia during home care, initial signs (e.g., coughing during meals, unintentional weight loss, reduced appetite) are often first observed by caregivers or during routine visits by home care providers. Periodic screening using EAT-10, the Saku-Saku Test, or simple water swallow tests is essential to detect early-stage dysphagia. Once suspected, an interdisciplinary team should be engaged promptly to evaluate risks, adjust diet texture, and initiate rehabilitative interventions such as oral motor exercises or breath–swallow coordination training. This approach helps prevent aspiration pneumonia and nutritional deterioration, which are common reasons for hospital readmission.

Management associated with HF involves combining swallowing rehabilitation, meal texture adjustments, oral care, and HF treatment itself. Swallowing rehabilitation typically includes tongue, pharyngeal, and neck muscle strengthening exercises, sensory stimulation training, and vocalization and breathing exercises. Depending on the patient's condition, individualized programs are developed considering circulatory factors, such as blood pressure elevation (43, 44).

In the nutritional management of dysphagia and HF, maintaining nutritional status is undoubtedly the foundation of treatment. When malnutrition and sarcopenia progress to advanced stages, the atrophy of the myocardium and the entire skeletal muscle system accelerates, often exacerbating dysphagia (19). In older patients with HF, even with similar levels of cardiac dysfunction, substantial differences in prognosis have been observed based on nutritional status (45, 46). Therefore, properly evaluating and addressing dysphagia at the earliest stage prevents aspiration and directly contributes to improving the overall outcomes of HF.

In the nutritional management of patients with HF, the basics typically include sodium restriction, fluid management, balancing potassium and minerals, and ensuring an adequate amount of energy intake. Excessive salt intake can lead to increased blood pressure and fluid retention, increasing the risk of acute exacerbations. Hence, salt restriction is a crucial strategy (47, 48). However, it was recently suggested that excessive salt and fluid restriction in older patients may lead to reduced appetite, malnutrition, and exacerbated frailty (49). Regarding dietary restrictions, protein intake is now also a focus. While caution is needed in patients with renal dysfunction, a growing view is that strict limitations are not necessarily required in cases of mild to moderate renal impairment, highlighting the need for individualized care (50, 51). Reportedly, adequate protein intake (approximately 1.2–1.6 g per kg of body weight per day) contributes to the prevention of sarcopenia in older adults (52). Regarding carbohydrate restriction, unless there are complications such as diabetes, the effect on long-term prognosis in older patients may not be clear. In fact, restrictions that lead to reduced activity levels or social participation are discouraged (53). Additionally, findings from epidemiological studies reveal that social networks and communication have a stronger influence on mortality and QOL than does dietary restrictions. Therefore, it is considered that excessive nutritional restrictions in HF management can increase the risk of lowering the patient's QOL and motivation (54, 55).

Studies show that serum albumin levels, which are strongly influenced by chronic inflammation and disease activity, may not always accurately reflect nutritional status (56). Therefore, in the nutritional assessment of older patients with HF, it is recommended to use a multidimensional approach. This should include evaluating albumin levels and indicators such as muscle mass (e.g., upper arm circumference measurement or bioelectrical impedance analysis), weight changes, and food intake, while carefully considering limb edema (57). Similarly, a multifaceted approach aimed at improving sarcopenia should be considered when implementing nutritional interventions. In addition, protein, sodium, and fluid intake should be adjusted individually based on renal function and electrolyte balance.

Adjusting meal texture includes the use of thickening agents, the introduction of pureed foods, the provision of soft foods, and strategies to reduce bite size. For patients with HF, consuming large amounts of food at once can significantly increase circulatory load. Therefore, setting smaller, more frequent meals can reduce the swallowing burden and help alleviate cardiac strain (58). Additionally, modifying posture during meals can promote airway protection and prevent aspiration. Specifically, a reclined position of 30° or the chin-down position (with the chin tucked) can support the function of the epiglottis (59, 60).

The oral health interventions in people with dysphagia are varied and lack clear guidelines (61). Typically, these interventions include a combination of instructions for effective tooth brushing or denture cleaning techniques and the use of a mouth rinse. As impaired swallowing function and oromotor inadequacies in patients with dysphagia may result in inadvertent aspiration of fluids and/or particles during dental procedures, the interventions are typically provided by oral care professionals, and ensuring regular dental check-ups is important (62). However, oral care intensity may vary across nursing home settings depending on staffing, training, and local care systems, and may be less intensive in some contexts (63).

In HF management, fluid control using diuretics and hemodynamic improvement have been suggested in several reports to alleviate swallowing disorders (64). In some cases, a reduction in left atrial pressure and improvement in pulmonary congestion have led to a decrease in the risk of aspiration (65). Such a multifaceted approach is necessary, and in home care, it is particularly important for the primary care physician, home care nurses, speech-language pathologists, dietitians, and dentists to collaborate, regularly assess the patient's condition, and respond flexibly while sharing information.

As heart failure progresses, goals of care often shift from disease optimization and prevention of exacerbations toward symptom relief, functional maintenance, and ultimately comfort-focused decision-making. Accordingly, dysphagia management in home care should be tailored to the disease stage and patient priorities, balancing aspiration risk with nutrition/hydration goals, medication feasibility, caregiver burden, and quality of life. This reframing provides the rationale for the end-of-life approach discussed in the next section, where shared decision-making and advance care planning become central to aligning feeding strategies with patient values.

## 6 End-of-life care and ethical considerations

In advanced heart failure, the question often shifts from “How can we prevent aspiration at all costs?” to “What feeding approach best aligns with the patient's goals and comfort?”

As severe HF (equivalent to New York Heart Association class III-IV or American College of Cardiology/American Heart Association stages C-D) progresses, a palliative approach in which QOL is prioritized is recommended owing to the limited prognosis (66, 67). At this stage, if dysphagia worsens and oral intake becomes difficult, the suitability of enteral nutrition and artificial life-sustaining treatments should be carefully discussed between healthcare providers, the family, and the patient. In patients with advanced dementia or end-stage heart failure, life expectancy is limited, and severe impairments in consciousness and swallowing function increase the risk of complications such as infections. In such cases, enteral nutrition may not necessarily improve quality of life. Alternative approaches may include maintaining oral intake as much as possible, prioritizing comfort-oriented oral care, and considering minimal hydration to relieve suffering. These options are supported both by clinical evidence and ethical considerations, and should be discussed with patients and families to ensure care aligns with their goals and values. During the end-of-life phase, feeding can impose a significant psychological and physical burden on caregivers due to the high risk of aspiration and choking. Healthcare professionals are also required to closely monitor intake and respond to emergencies, which increases the demand for medical resources and staff workload. These efforts may not always lead to improvements in the patient's quality of life, making it essential to reevaluate the meaning and goal of oral intake based on the patient's wishes and overall condition. Thus, there is concern that excessive nutritional management could potentially increase suffering and the burden of medical and caregiving efforts (68).

On the other hand, if a patient expresses the desire to eat even a little orally, it is important to respect this choice, explain the risks of eating, and consider the balance between the risk of aspiration pneumonia and the joy of eating. Continuing oral intake, even with the potential risks, can be considered as one option (8). In situations where complete prevention of aspiration is difficult, it is recommended to prioritize the patient's wishes and dignity and to provide opportunities to eat small amounts of their preferred food. Decision-making regarding end-of-life care in these situations is ethically complex. Additionally, it is essential to hold regular conferences within a multidisciplinary team to share information and support decision-making (69).

Furthermore, the concepts of advance care planning and living wills are becoming more widespread.

Accordingly, early discussions in home care are increasingly encouraged for patients with HF regarding preferred treatments and care as their condition progresses (70, 71). Through this process, discrepancies in understanding between the patient, family, and healthcare providers may be reduced.

**Figure 2:**
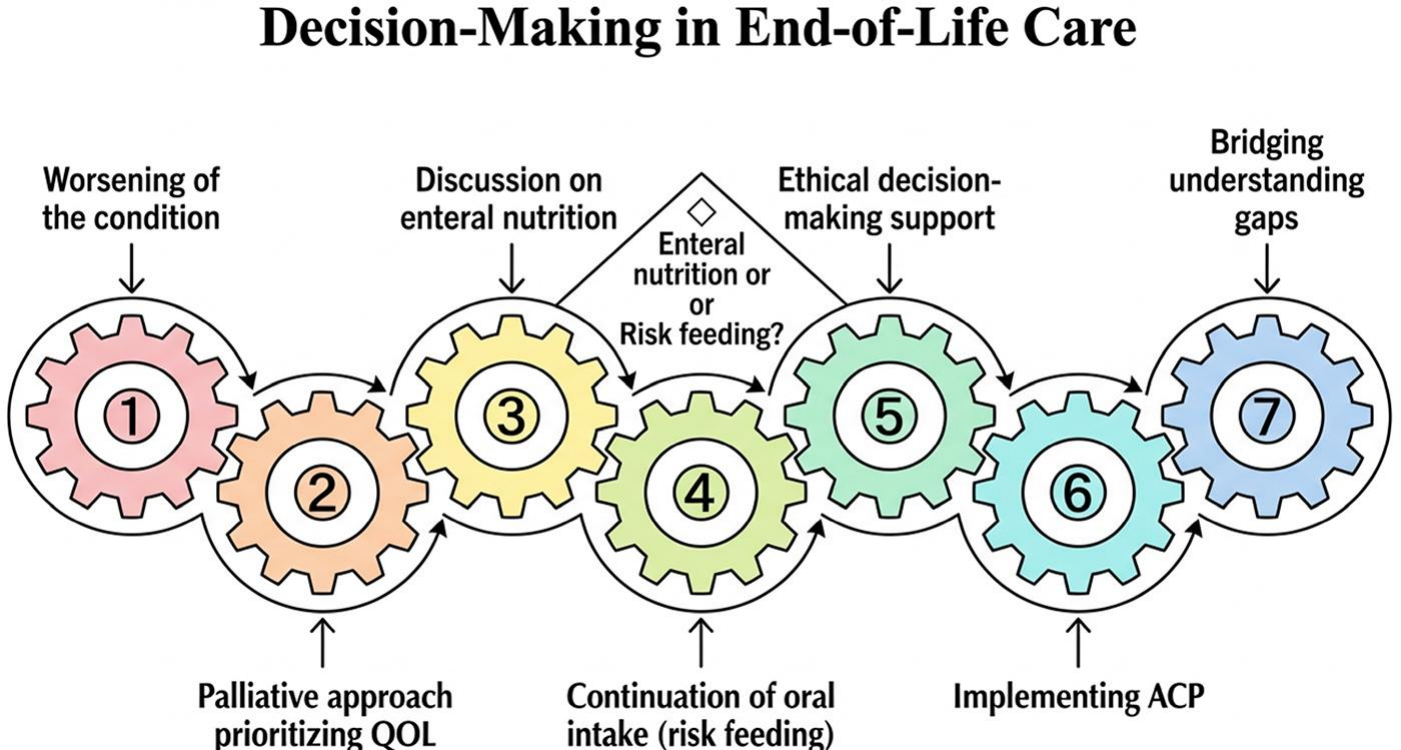
Decision-Making in end-of-life care. The interlocking gears depict an iterative decision-making process for end-of-life dysphagia care in homebound older adults with HF. The numbered gears represent interdependent domains that are revisited as the condition changes, including a palliative approach prioritizing quality of life (QOL), discussions on enteral nutrition, continuation of oral intake with acknowledged risks (“risk feeding”), ethical decision-making support, implementing advance care planning (ACP), and bridging understanding gaps among the patient, family, and care team. The central diamond highlights the recurring decision point between enteral nutrition and risk feeding, which should be aligned with goals of care and patient/family preferences. ACP, advance care planning; QOL, quality of life.

This can provide a clearer view of the medical measures and caregiving burden associated with progression of HF and swallowing disorders (Figure 2).

## 7 Limitations

The integrated approach to evaluate and manage HF and dysphagia has potential usefulness based on findings from recent studies and clinical reports; nevertheless, there is still no fully established evidence. Large-scale intervention studies and longitudinal research supporting this approach are still lacking. For instance, the effects of swallowing rehabilitation on reducing readmission rates in patients with HF, the impact of thorough oral care on improving prognosis, and cost-effectiveness remain unresolved issues. Particularly, research focused on older patients who are homebound is scarce, and it is important to build a systematic body of practical knowledge through case reports and small-scale studies. In addition, variations in regional healthcare systems and home care practices affect the generalizability of the findings. Future studies should aim to accumulate case-based evidence and adopt standardized outcome measures to strengthen this integrated care approach. Several foundational estimates cited in this review are derived mainly from hospital-based observational studies. Direct evidence focused on homebound populations remains limited; therefore, some recommendations should be adapted to local home care resources and interpreted cautiously.

## 8 Conclusion

Integrating an HF-focused perspective into the management of dysphagia in older patients who are homebound could be a key factor that significantly impacts their prognosis and QOL. Circulatory failure, congestion, sarcopenia, and dyspnea caused by HF can weaken swallowing function, while dysphagia-related malnutrition and aspiration pneumonia can worsen HF, creating a negative cycle. By considering the pathophysiological connections and conducting appropriate assessments and interventions, it will be possible to support patients and families in various ways, such as maintaining oral intake, preventing readmission, and focusing on QOL in end-of-life care. Particularly, in home care settings, even when specialized testing is difficult, combining meal rounds and simple screening methods (RSST, modified water swallow test, EAT-10, and the Saku-Saku Test) and early sharing of problems through a multidisciplinary team is crucial.
